# A High-Speed Congenic Strategy Using First-Wave Male Germ Cells

**DOI:** 10.1371/journal.pone.0004943

**Published:** 2009-03-31

**Authors:** Narumi Ogonuki, Kimiko Inoue, Michiko Hirose, Ikuo Miura, Keiji Mochida, Takahiro Sato, Nathan Mise, Kazuyuki Mekada, Atsushi Yoshiki, Kuniya Abe, Hiroki Kurihara, Shigeharu Wakana, Atsuo Ogura

**Affiliations:** 1 RIKEN BioResource Center, Tsukuba, Ibaraki, Japan; 2 Graduate School of Life and Environmental Science, University of Tsukuba, Tsukuba, Ibaraki, Japan; 3 Department of Physiological Chemistry and Metabolism, Graduate School of Medicine, The University of Tokyo, Bunkyo-ku, Tokyo, Japan; 4 Center for Disease Biology and Integrative Medicine, Graduate School of Medicine, The University of Tokyo, Bunkyo-ku, Tokyo, Japan; 5 Tsukuba Safety Assessment Laboratories, Banyu Pharmaceutical Company Limited, Tsukuba, Ibaraki, Japan; Buck Institute for Age Research, United States of America

## Abstract

**Background:**

In laboratory mice and rats, congenic breeding is essential for analyzing the genes of interest on specific genetic backgrounds and for analyzing quantitative trait loci. However, in theory it takes about 3–4 years to achieve a strain carrying about 99% of the recipient genome at the tenth backcrossing (N10). Even with marker-assisted selection, the so-called ‘speed congenic strategy’, it takes more than a year at N4 or N5.

**Methodology/Principal Findings:**

Here we describe a new high-speed congenic system using round spermatids retrieved from immature males (22–25 days of age). We applied the technique to three genetically modified strains of mice: transgenic (TG), knockin (KI) and *N*-ethyl-*N*-nitrosourea (ENU)-induced mutants. The donor mice had mixed genetic backgrounds of C57BL/6 (B6)∶DBA/2 or B6∶129 strains. At each generation, males used for backcrossing were selected based on polymorphic marker analysis and their round spermatids were injected into B6 strain oocytes. Backcrossing was repeated until N4 or N5. For the TG and ENU-mutant strains, the N5 generation was achieved on days 188 and 190 and the proportion of B6-homozygous loci was 100% (74 markers) and 97.7% (172/176 markers), respectively. For the KI strain, N4 was achieved on day 151, all the 86 markers being B6-homozygous as early as on day 106 at N3. The carrier males at the final generation were all fertile and propagated the modified genes. Thus, three congenic strains were established through rapid generation turnover between 41 and 44 days.

**Conclusions/Significance:**

This new high-speed breeding strategy enables us to produce congenic strains within about half a year. It should provide the fastest protocol for precise definition of the phenotypic effects of genes of interest on desired genetic backgrounds.

## Introduction

For nearly 30 years, the genetic manipulation of laboratory mice has contributed substantially to the development of many fields in medical research and mammalian biology. Remarkably, by genetically altering the mouse genome with single nucleotide precision, it is now possible to create mice with desired genetic modifications to assess gene function in healthy animals and in animal models for human diseases. One major issue associated with mouse genetic engineering is that the biological function of engineered genes can vary with their genetic background [1]–[3]. This often raises serious concerns, because transgenic (TG) or knockout (or knockin, KI) mice are generated in strains that have historically been selected for the ease and convenience of generating the TG or knockout strain, rather than phenotypic characterization of the mutation itself. For example, most embryonic stem (ES) cell lines used for knockout experiments are derived from the 129 strain. Unfortunately, however, this strain has significant biological limitations that interfere with the phenotypic analysis of a target mutation. It consists of a diverse and complex family of substrains [4] and many of these have an atypical brain structure [5]. Therefore, for facilitating definition of transgene or gene-targeted effects over a given genetic background, the engineered gene should be introduced from the donor strain into the desired recipient strain.

The classical protocol for such purpose is congenic breeding: serially backcrossing the gene donor to the recipient strain accompanied by selection for progeny carrying the desired gene in each backcross generation. This protocol calls for 10 backcross generations (N10), followed by an intercross (F1) to produce founders that are homozygous for the desired gene (theoretically more than 99% of the genome) [6]. Although the strategy is simple, the process is expensive and time consuming, requiring roughly 3–4 years to produce any given congenic strain. To overcome this weakness, reduction of backcross generations for the establishment of congenic strains has been achieved using marker-assisted selection protocols (MASP), the so-called ‘speed congenics’. The time required for deriving such congenic strains is about 1–2 years, depending on the robustness and intensiveness of the polymorphic analysis between the gene donor and recipient strains [7].

One interesting suggestion is that the breeding cycle could be shortened by superovulating and breeding juvenile females (3–4 weeks) followed by embryo transfer to mature females for production of the next generation [8]. This might shorten the generation time down to 6–7 weeks and reduce the whole congenic procedure to 1 year. This ‘supersonic congenics’ strategy was promising, but has not proved practical because of the limited number of oocytes that can be produced and because there are great individual differences in response to superovulation resumes.

We have attempted to develop another high-speed congenic strategy through the male germline. Recently, we have shown that the genomes of male germ cells from the first wave of spermatogenesis have the ability to support embryonic development to term. Mouse round spermatids—the youngest haploid male germ cells—appear first at 17 days after birth and can be used for the production of offspring by round spermatid injection (ROSI) into oocytes [9]. We applied this technique to the generation of congenic strains from mice with mixed genotypes bearing a transgene, a targeted KI gene or chemically induced mutant genes. At each generation, males used for backcrossing were selected based on polymorphic marker analysis: low density screening MASP using 74–176 markers distributed uniformly throughout the genome. The recipient strain for the expected genetic background was C57BL/6 for all lines of congenics. The results were very consistent and the time for producing a congenic strain was reduced significantly. Therefore, our high-speed congenic system would be very useful for the accelerated analysis of genes of interest under a defined genetic background.

## Results and Discussion

### Definition of the optimal male age for spermatid collection

**Figure 1:**
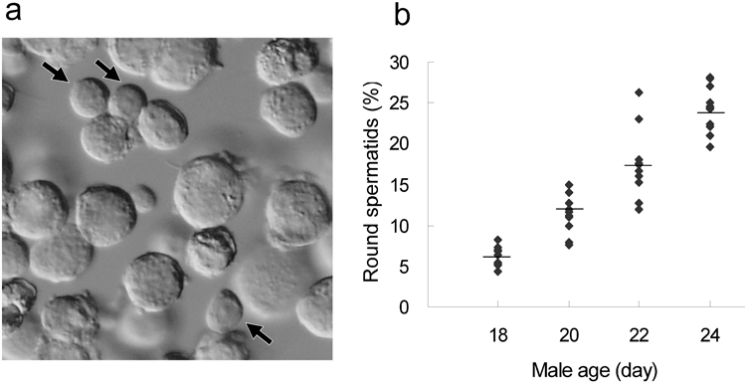
Definition of the optimal male age for spermatid collection. a) Representative photomicrograph of a cell suspension prepared from the testis of a male mouse at 24 days of age. Arrows indicate round spermatids, which are easily identified by a round nucleus and a high cytoplasmic/nuclear ratio. b) The proportion of round spermatids among testicular cells from 18 days to 24 days after birth. The percentages of round spermatids in testicular suspension increased consistently from days 18 to 24 (P<0.05 between groups). The cells were counted in two different males by two different operators. The horizontal bars indicate the average.

In mice, round spermatids can be collected from 17-day-old males at the earliest and their genomes can support full term embryonic development after injection into oocytes using ROSI [9]. However, the efficiency of producing offspring using these round spermatids was extremely low (0.9%) because of their very low incidence in testicular cell suspensions (<2%). This might compromise the accurate identification of round spermatids within a limited time of oocyte micromanipulation. Therefore, we first checked the proportion of round spermatids in testicular cell suspensions from males aged 18, 20, 22 and 24 days to define the optimal age for applying ROSI. As shown in Figure 1, the percentages of round spermatids in testicular suspension increased consistently from days 18 to 24 with statistically significant differences between groups (P<0.05). This resulted in easier identification of round spermatids under a microscope: thus, the time required for picking up a single round spermatid was roughly 60, 15, 10 and 10 sec using cell suspensions collected at days 18, 20, 22 and 24, respectively. Therefore, we defined day 22 to be the earliest age of males that allowed the efficient identification of round spermatids in testicular cell suspensions. Testes of the mice at day 22 were smaller than in adults, but we could still collect sufficient round spermatids from a single testis to perform a ROSI experiment (about 150–250 injected oocytes).

### Congenics of gene-modified strains using first-wave round spermatids

To test whether high-speed congenics using the first wave of round spermatids could be used practically, we applied the technique to three different types of gene-modified strains, TG, KI and *N*-ethyl-*N*-nitrosourea (ENU)-mutant strains. At each generation, a male used for the next application of ROSI was selected based on showing fewer heterozygous alleles by polymorphic markers that could identify the donor (DBA/2 and 129) and recipient strains (B6Cr and B6J). Backcross ROSI was repeated until the N4 or N5 generation. After this, additional backcrossing was continued by natural mating to reduce the undetected gaps of contaminating donor alleles [10].

**Figure 2:**
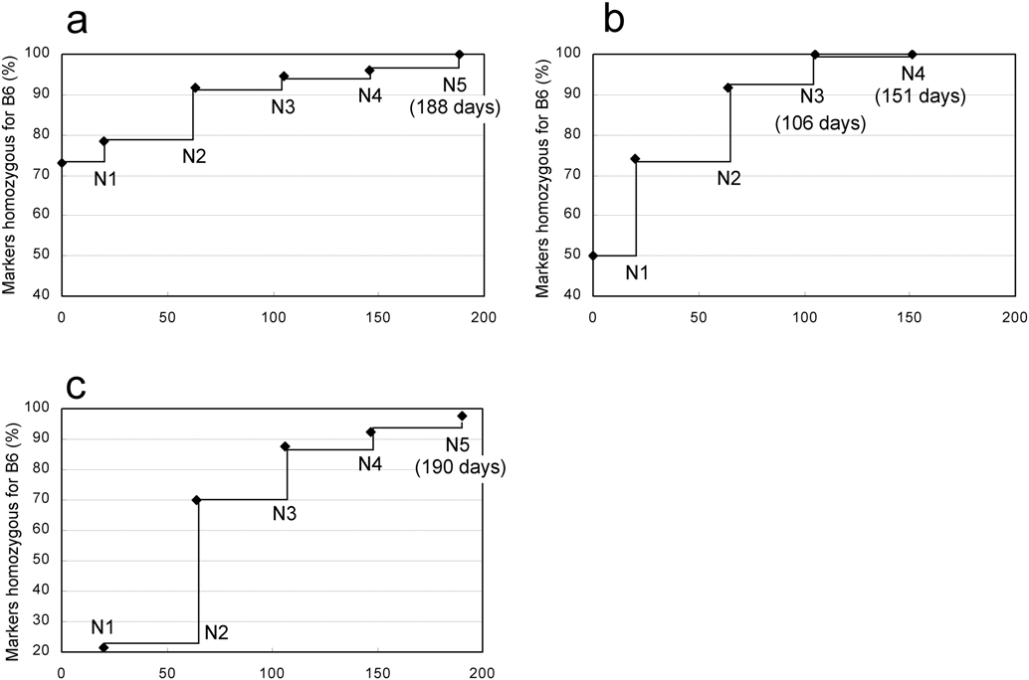
Time course of generation turnover and the rate of markers homozygous for the C57BL/6 (B6) type. a) *Vasa–Venus* transgenic strain. All markers (n = 74) were homozygous for B6 at N5 on day 188. b) *Ednra^EGFP/+^* knockin strain. All markers (n = 86) were homozygous for B6 at N3 on day 106 and N4 offspring were obtained on day 151. c) ENU-induced *Gdf5* mutant strain. The N5 generation was obtained on day 190 and was 97.7% (172/176) homozygous for B6. There were 74, 86 and 176 polymorphic markers, which identified the alleles for the C57BL/6Cr:DBA/2Cr, C57BL/6Cr:129 and C57BL/6J:DBA/2J strains, respectively. Each generation turnover was between 42 and 45 days: the age of the donor male plus the gestation period (20 days) minus the one-day overlap between them.

The *Vasa–Venus* TG strain we used was generated from embryos produced by IVF using (B6Cr×DBA/2)F1 strain oocytes and B6Cr strain spermatozoa, and maintained by full-sib mating. The N1 offspring were obtained by ICSI using a donor male at F7. As shown in Figure 2a, N5 backcross offspring were obtained on day 188 and all 74 markers were identified as B6 homozygous in one of two carrier males.

The *Ednra^EGFP/+^* KI strain was derived from ES cells with a (B6Cr×129*^+Ter^*/SvJcl)F1 genetic background [11]. The first N1 generation was obtained by IVF using B6Cr oocytes and spermatozoa from a chimeric mouse. All 86 markers were homozygous for B6Cr as early as at N3 (day 106; 2 out of 14 carrier males) and N4 offspring were obtained on day 151 (Figure 2b).

The ENU-induced growth differentiation factor 5 (*Gdf5*) mutant line had a mixed genetic background of B6J and DBA/2J [12]. For this combination of inbred strains, more dense polymorphic markers were available using single nucleotide polymorphism (SNP) assays as well as microsatellite genotyping (176 markers; see Materials and Methods). The N5 generation was obtained on day 190 and was 97.7% (172/176) homozygous for B6J (Figure 2c). The following N6 and N7 generations produced by IVF were 98.8% and 99.4% homozygous for B6J, respectively.

The efficiency rates in backcross breeding by ROSI in these gene-modified strains are shown in Table 1. All the modified genes could be propagated successfully into the next generations by ROSI. The male carriers finally obtained were all fertile and propagated the modified genes to the next generation by natural mating.

**Table 1:** Results of congenic breeding by round spermatid injection (ROSI) in three gene-modified strains.

Strain	Generation produced	Age (day) of male used for ROSI	No. of oocyes that survived ROSI	No. (%) of oocytes that developed to 2-cells	No. of 2-cells transferred	No. (%) implanted	No. (%) born	No. (%) of males born	No. (%) of male carriers
*Vasa-Venus* transgenic	N1	Adult (ICSI)	16	15 (93.8 )	15	10 (66.7 )	5 (33.3 )	5 (33.3 )	5 (33.3)*
	N2	24	182	164 (90.1 )	164	69 (42.1 )	24 (14.6 )	12 (7.3 )	4 (2.4 )
	N3	23	123	109 (88.6 )	109	24 (22.0 )	4 (3.7 )	2 (1.8 )	1 (0.9 )
	N4	22	172	156 (90.7 )	156	51 (32.7 )	14 (9.0 )	6 (3.8 )	2 (1.3 )
	N5	23	134	123 (91.8 )	123	23 (18.7 )	10 (8.1 )	4 (3.3 )	2 (1.6 )
*Ednra-EGFP* knockin**	N1	Adult (IVF)	166	135 (81.3 )	135	62 (45.9 )	49 (36.3 )	23 (17.0 )	12 (8.9 )
	N2	24	243	196 (80.7 )	196	63 (32.1 )	30 (15.3 )	10 (5.1 )	5 (2.6 )
	N3	24	261	235 (90.0 )	235	107 (45.5 )	52 (22.1 )	30 (12.8 )	14 (6.0 )
	N4	25	244	215 (88.1 )	215	72 (33.5 )	39 (18.1 )	19 (8.8 )	11 (5.1 )
ENU-induced mutant	N1	Adult (IVF)	80	49 (61.3 )	49	Not observed	26 (53.1 )	14 (28.6 )	3 (6.1 )
	N2	25	201	158 (78.6 )	158	54 (34.2 )	25 (15.8 )	11 (7.0 )	4 (2.5 )
	N3	23	208	194 (93.3 )	194	68 (35.1 )	26 (13.4 )	12 (6.2 )	7 (3.6 )
	N4	22	173	127 (73.4 )	127	51 (40.2 )	13 (10.2 )	5 (3.9 )	2 (1.6 )
	N5	24	216	177 (81.9 )	177	91 (51.4 )	34 (19.2 )	10 (5.6 )	4 (2.3 )
Total (ROSI only)			2157	1854 (86.0 )	1854	673 (36.3 )	271 (14.6 )	121 (6.5 )	56 (3.0 )
Control (C57BL/6)		22–24	467	349 (74.7 )	329	133 (40.4 )	49 (14.9 )	26 (7.9 )	

* The implantation sites were identified as the scars of decidualization at caesarian section.

** The donor male (N0) was homozygous for the transgene.

*** All genetic markers tested were homozygous for the B6 mouse strain at N3.

### Significance of congenic breeding using first-wave male germ cells

Congenic strains have been used extensively for the study of mouse genetics including definition of phenotypic effects of genes on specific genetic backgrounds and identification of genes or genomic segments affecting the phenotypes of interest by quantitative trait locus (QTL) analysis. However, it takes about 2–3 years to construct a congenic strain with a level of genetic homogeneity that is reliable for research (>99% or more) [6]. To accelerate congenic breeding, MASP has been developed by taking advantage of precise information on mouse genetics [7]. Another approach for efficient congenics should be rapid generation turnover by assisted reproduction techniques. The use of immature females proposed by Behringer [8] was applied successfully to the derivation of a conplastic strain of rats [13]. However, there have been very few similar applications published, probably because of the limited number of oocytes produced from any one female, which may attenuate the number of carrier females in the subsequent generations. Our high-speed congenic system uses immature males as founders at each generation. Unlike females, each male can produce a large number of germ cells and multiple litters of offspring. This not only assures the safe propagation of the target gene on to the next generation, but also enables efficient selection of a male for the next round of ROSI, thus significantly shortening the time for congenics.

In this study, the advantages of our high-speed congenic strategy were shown in the KI strain, which started with a (B6Cr×129)F1 genetic background: all 86 markers were homozygous for the B6 strain as early as 106 days (N3). Apparently, it is more straightforward to use B6 ES cells for gene targeting if the B6 genetic background is required. Several B6 ES cells are available for gene targeting [14], [15], and the International Knockout Consortium uses C57BL/6 ES cell lines [16]. However, B6 ES cells usually require more intense care than other 129 or hybrid ES cells to maintain their germline transmission ability during gene targeting. ES cells with hybrid genetic constitutions of B6 and 129 are easy to maintain and can be used efficiently for producing gene-targeted offspring [1], [11], especially by tetraploid complementation [17]. If the gene-targeted allele is of B6 origin, it may avoid the persistence of donor genetic segments around the targeted allele during congenic production, which inevitably occurs when 129 ES cells are used. Thus, the combination of gene-targeting (B6×129)F1 ES cells and the new high-speed congenic breeding may be an alternative fast protocol to generate a B6 gene-targeting strain.

A congenic breeding strategy has also been employed extensively in laboratory rats because it is now possible to map the genetic variants and mutations that underlie complex disease-related phenotypes in this species [18], [19]. ROSI is successful in some, but not all, strains of rats [20]. As the first wave of round spermatids appears in rat testes around 26 days after birth, we estimate that congenic rat strains could be generated within 7 or 8 months by using these germ cells [21].

We anticipate that the congenic strategy developed in this study might be accelerated further using male germ cells that are younger than round spermatids. This is possible theoretically, because the genomes of primary spermatocytes—premeiotic male germ cells—can support the full term development of embryos [22]. Despite many efforts to improve the technique, however, the efficiency of producing offspring using primary spermatocytes is very low [22], [23]. Therefore, at present the use of round spermatids on days 22–25 may be the most practical range for efficient, rapid backcross breeding in mice. As far as we know, this is the most rapid generational turnover by sexual reproduction in mammals.

### Technical issues associated with congenics by ROSI

As mentioned above, the high-speed congenic strategy we developed is very promising to produce congenic strains with desired genetic backgrounds. One of the technical issues associated with this strategy is that ROSI needs some skill and experience. However, ROSI is generally easier to perform than conventional ICSI because of the high survival rate of oocytes after injection: the diameter of injection pipettes is small and the activated oocytes used for ROSI are more resistant to the injection stimulus than nonactivated oocytes [24]. From our experience, training of three to four consecutive weeks is enough for ROSI if the operator already has the basic technique for embryo handling. For ICSI, reliable protocol papers are available [25], [26] and the same protocols can be essentially applied to ROSI. Oocytes from B6 females tend to be more sensitive to injection than those from other strains, but this problem might be overcome by using a high osmotic strength medium for manipulation on the microscope stage, if necessary. In our ROSI experiments using B6 oocytes, about 80% survived the injection whereas about 90% survived in other strains including B6D2F1, DBA/2 and 129 (unpublished).

As shown in Table 1, we consistently obtained sufficient carrier males for selection except for the N3 to N5 generations in the *Vasa–Venus* TG strain, which were affected by an accidental decline in the quality of recipient females, for reasons unknown. Based on the overall efficiency in our ROSI experiments presented in Table 1, we estimated the number of oocytes to be injected with the aim of obtaining expected number of carrier males (Table 2). These numbers of oocytes can be handled by one or two operators in a single session.

**Table 2:** The numbers of superovulated females and oocytes required for obtaining selectable numbers of carrier males, as estimated from the data in Table 1.

Females superovulated (25–30 oocytes per female)	4 to 5	6 to 7	7 to 8
Oocytes injected	125	167	208
Oocytes survived (80% per oocytes injected)	100	133	167
2-cells transferred (80% per oocytes survived)	80	107	133
Birth (15% per 2-cells transferred)	12	16	20
Males (50% per birth)	6	8	10
Carrier males (50% per males)	3	4	5

One can question if epigenetic modifications might have occurred during conception using round spermatids, because ROSI-derived preimplantation embryos have shown some disturbances in gene expression [27], [28] and aberrant DNA methylation [29]. However, epigenetic errors imposed on individuals are normally erased during germ cell development and are never transmitted to the next generation by natural mating, as shown in mouse somatic cell cloning experiments [30], [31]. Therefore, once mated naturally, congenic strains produced by ROSI are expected to become epigenetically indistinguishable from those produced by conventional congenic protocols.

### Conclusions

The generation turnover time in mice can be shortened to about 40 days by using the first wave round spermatids as male gametes. We confirmed that this breeding strategy reduced the time required for congenesis to about half a year. This should provide the earliest opportunities for the analysis of genes of interest under a defined genetic background and for QTL mapping, which are becoming integral to biomedical research using the mouse as a model.

## Materials and Methods

### The origin of donor strains

The B6 substrains used in this study were B6Cr (C57BL/6CrSlc) and B6J (C57BL/6JJcl), which were purchased from CLEA Japan, Inc. (Kanagawa) and Japan SLC, Inc. (Shizuoka), respectively [32]. One mature male mouse from each strain was used as the donor of the modified gene. The TG strain we used was Tg(Mvh-Venus)1Rbrc, which was generated by DNA nuclear injection into zygotes derived from IVF using (B6Cr×DBA/2)F1 oocytes and B6Cr spermatozoa. The *Mvh-Venus* gene clearly shows the germline origin of living cells by green fluorescence because of the highly specific expression of the *Mvh* (mouse vasa homologue) gene [33]. The strain was maintained by full-sib mating. An F7 male homozygous for the transgene was used as the donor.

The KI line we used was the *Ednra^EGFP/+^* strain carrying the reporter gene for enhanced green fluorescence protein (EGFP) that had been knocked into the *Ednra* (endothelin receptor type A) locus by recombinase-mediated cassette exchange based on the Cre-lox system [11]. The gene-targeted ES cells had the (B6Cr×129*^+Ter^*/SvJcl)F1 genotype. Chimeric embryos were produced by injection of ES cells into ICR blastocysts and they were transferred into pseudopregnant ICR females. A chimeric male thus obtained were used for producing N1 by conventional IVF using B6Cr oocytes.

The ENU-induced mutant strain we used carried a point mutation at the *Gdf5* locus. This mutation causes an amino acid substitution in a highly conserved region of the active signaling domain of the GDF5 (growth differentiation factor 5) protein, leading to impaired joint formation and osteoarthritis [12]. The donor male had a mixed genetic background of B6J and DBA/2 because the strain was generated from a cross of an ENU-mutagenized B6J male and a wild-type DBA/2 female.

Offspring that carried modified genes were genotyped at each generation by polymerase chain reaction (PCR) amplification with specific primers for the given TG strain [34], by specific green fluorescence over the body for the KI strain and by PCR-based sequencing for the ENU mutant strain [12].

### Collection of oocytes

Female B6Cr or B6J strain mice (7–10 weeks old) were each injected with 7.5 units of equine chorionic gonadotropin followed by injection of 7.5 units of human chorionic gonadotropin (hCG) 48 h later. Mature oocytes were collected from the oviducts 15–17 h after hCG injection and were freed from cumulus cells by treatment with 0.1% hyaluronidase in CZB medium [35]. The oocytes were transferred to fresh CZB medium and incubated at 37°C in an atmosphere of 5% CO_2_ in air for up to 90 min before ROSI.

### Preparation of testicular cell suspensions

Spermatogenic cells were prepared mechanically as described for hamsters [36]. Briefly, testes were removed from 18- to 25-day-old males and placed in erythrocyte-lysing buffer (155 mM NH_4_Cl/10 mM KHCO_3_/2 mM EDTA, pH 7.2). For the first series of experiments to identify the optimal age of males for donors, we used ICR males; as far as we examined there were no strain-dependent differences in the timing of the first wave of spermatogenesis. The tunica albuginea was removed and the seminiferous tubule masses were transferred into cold (4°C) Dulbecco's phosphate buffered saline (PBS) supplemented with 5.6 mM glucose, 5.4 mM sodium lactate and 0.1 mg/ml of polyvinyl alcohol (polyvinylpyrrolidone, PVP, in the original report) (GL-PBS) [36]. The seminiferous tubules were cut into small pieces and pipetted gently to disperse spermatogenic cells into the GL-PBS. Then, the cell suspension was filtered through a 38-µm nylon mesh and washed three times by centrifugation (200 *g* for 4 min). To define the optimal male age for spermatid collection, we first examined the proportion of round spermatids in cell suspensions collected from two males aged at 18, 20, 22, or 24 days. The percentages of round spermatids were analyzed using arcsine transformation, followed by one-way ANOVA analysis and a *post-hoc* procedure using Scheffe's test for multiple comparisons.

### ROSI

ROSI was performed using a Piezo-driven micromanipulator (Prime Tech Ltd., Ibaraki, Japan) as described [37], [38]. The cover of a plastic dish (Falcon no. 1006; Becton Dickinson, Franklin Lakes, NJ) was used as a microinjection chamber. Several small drops (∼4 µl) of Hepes-buffered CZB with or without 10% PVP were placed on the bottom and covered with mineral oil. Spermatogenic cells were placed in one of the PVP droplets. Before injection of the nuclei of round spermatids, oocytes were activated by treatment with Ca^2+^-free CZB medium containing 2.5 mM SrCl_2_ for 20 min at 37°C. Oocytes reaching telophase II at 40–90 min after onset of activation treatment were each injected with a round spermatid. They were kept in Hepes-CZB at room temperature (24°C) for ∼10 min before culture in CZB at 37°C under 5% CO_2_ in air.

### Embryo culture and transfer

Embryos that reached the 2-cell stage by 24 h of culture in CZB were transferred into the oviducts of pseudopregnant ICR strain females (8–12 weeks old) on the day after mating (day 0.5). On day 19.5, recipient females were killed and their uteri were examined for live term fetuses. These were nursed by lactating ICR foster females. The day of birth was designated day 0.5 for newborns.

### Care and use of animals

All procedures described here were reviewed and approved by the Animal Experimental Committee at the RIKEN Institute.

### Genotyping for MASP

Tail clips about 0.3 cm long were collected for DNA extraction, using the Wizard Genomic DNA Purification Kit (Promega, Madison, WI) and the DNeasy 96 Blood & Tissue Kit (#69582; QIAGEN GmbH, Hilden, Germany) according to the manufacturers' instructions. Microsatellite genotyping was carried out by PCR for simple sequence length polymorphisms (SSLP) using microsatellite markers. The microsatellite markers were selected out of sequence length polymorphisms between B6 and DBA/2 and B6 and 129 strains (Mekada et al., unpublished data) (Tables S1 and S2). PCR execution was performed using the QIAGEN Multiplex PCR kit (#206143; QIAGEN GmbH) and the length polymorphism was detected by agarose gel electrophoresis. Map locations and primer sequences of the microsatellite loci were used according to the Mouse Genome Informatics (MGI) of the Jackson Laboratory, USA, and Mouse Microsatellite Data Base of Japan (MMDBJ).

SNP genotyping was carried out using a TaqMan Minor Groove Binding (MGB) assay (Applied Biosystems, Foster City, CA). TaqMan MGB probe sets were designed based on SNP polymorphism between C57BL/6J and DBA/2J (Table S3). PCR execution was performed using TaqMan Genotyping Master Mix (#4371353; Applied Biosystems). SNP polymorphisms were detected using an ABI PRISM 7900HT Sequence Detection System (Applied Biosystems).

## Supporting Information

Table S1 Variation alleles between C57BL/6 and DBA/2 mouse strains.
(0.02 MB XLS)

Table S2 Variation alleles between C57BL/6 and 129 mouse strains.
(0.02 MB XLS)

Table S3 The SNPs information that designed the TaqMan MGB probe sets.
(0.02 MB XLS)
